# Homeostatic regulation of adult hippocampal neurogenesis in aging rats: long-term effects of early exercise

**DOI:** 10.3389/fnins.2014.00174

**Published:** 2014-07-01

**Authors:** Christina M. Merkley, Charles Jian, Adam Mosa, Yao-Fang Tan, J. Martin Wojtowicz

**Affiliations:** Department of Physiology, Faculty of Medicine, University of TorontoToronto, ON, Canada

**Keywords:** adult neurogenesis, exercise, aging, hippocampus, dentate gyrus, plasticity, homeostasis

## Abstract

Adult neurogenesis is highly responsive to environmental and physiological factors. The majority of studies to date have examined short-term consequences of enhancing or blocking neurogenesis but long-term changes remain less well understood. Current evidence for age-related declines in neurogenesis warrant further investigation into these long-term changes. In this report we address the hypothesis that early life experience, such as a period of voluntary running in juvenile rats, can alter properties of adult neurogenesis for the remainder of the animal's life. The results indicate that the number of proliferating and differentiating neuronal precursors is not altered in runners beyond the initial weeks post-running, suggesting homeostatic regulation of these processes. However, the rate of neuronal maturation and survival during a 4 week period after cell division was enhanced up to 11 months of age (the end of the study period). This study is the first to show that a transient period of physical activity at a young age promotes changes in neurogenesis that persist over the long-term, which is important for our understanding of the modulation of neurogenesis by exercise with age. Functional integration of adult-born neurons within the hippocampus that resist homeostatic regulation with aging, rather than the absolute number of adult-born neurons, may be an essential feature of adult neurogenesis that promotes the maintenance of neural plasticity in old age.

## Introduction

With aging, levels of adult neurogenesis in the dentate gyrus (DG) show a precipitous decline, evident in a variety of mammalian species (Seki and Arai, 1995; Cameron and McKay, 1999; Gould et al., 1999; Kempermann et al., 2002; McDonald and Wojtowicz, 2005). This effect does not appear to be due to a relatively impoverished environment experienced by laboratory rats and mice (Epp et al., 2009), but is well-conserved. Declining levels of adult neurogenesis with age have prompted speculation as to the functional and physiological relevance of these new neurons (Snyder and Cameron, 2012). However, an increasing number of studies support the contention that adult neurogenesis is involved in certain types of learning behavior and memory (Abrous et al., 2005). Specifically, memories relying on pattern separation in the DG, the ability to transform highly similar experiences into discrete and non-overlapping representations (Kesner, 2007; Clelland et al., 2009; Sahay et al., 2011), and those that are prone to interference (Appleby and Wiskott, 2009; Aimone et al., 2011), depend on the number of adult-born dentate granule cells. The enhanced plasticity that is a characteristic of newly formed dentate granule cells (Snyder et al., 2001), is likely critical for the maintenance of cognitive performance throughout life and into old age.

Aging is associated with cognitive decline in both rodent and human subjects (Foster, 1999), and it is tempting to speculate that reduced hippocampal neurogenesis is partially responsible for the cognitive changes with aging (Lazarov et al., 2010; Mu and Gage, 2011; Galea et al., 2013). This idea is supported by rodent studies, in which age-related cognitive decline is associated with decreased neurogenesis (Kempermann et al., 2002; Van Praag et al., 2005; Creer et al., 2010). However, early data on rodents in the wild (Barker et al., 2005; Epp et al., 2009), and recent data in humans (Spalding et al., 2013) suggest that the magnitude of age-related declines may not be the same across all species. Thus, a better understanding of the life-long trajectory of neurogenic decline and its regulation is warranted.

Physical exercise is a well established stimulator of neurogenesis (Van Praag, 2008), and has been shown to enhance cognitive performance along with increasing neurogenesis (Kempermann et al., 2002; Van Praag et al., 2005; Leal-Galicia et al., 2008; Wojtowicz et al., 2008; Creer et al., 2010). Modulating neurogenesis via physical exercise may play an important role in the prevention of age-related cognitive decline (Galea et al., 2013). One primary question in the current study is whether early exposure to physical exercise can exert long-term effects on neurogenesis, which may delay or alter the trajectory of age-related declines.

In humans, it is well documented that age-related cognitive decline is linked to the intellectual history of individuals, and several studies have confirmed an influence of early age education, physical exercise, or adult occupational achievements on cognitive performance in healthy subjects, and on the rate of cognitive decline with disease (Snowdon, 1997; Sibley and Etnier, 2003; Andel et al., 2008; Åberg et al., 2009; Mortimer, 2012; Puccioni and Vallesi, 2012). It has been hypothesized that these effects are due to a “cognitive reserve” developed early on that can buffer individuals from the consequences of Alzheimer's or dementia (Stern, 2002), although the biological bases for cognitive reserve are largely unknown. Furthermore, the mechanisms that underlie a sustained influence of brain activity at a young age on the brain's performance during cognitive decline remain elusive. In recent years, the “Neurogenic Reserve Hypothesis” has emerged, postulating that activity-dependent maintenance of neurogenesis creates a pool of recruitable cells which persists into old age and can thereby elicit greater capacity for enduring plasticity and adaptability (Kempermann, 2008).

In the present study, we are addressing the neurogenic reserve hypothesis as it relates to long term alterations in hippocampal neurogenesis. This study has been guided by recent findings showing that adult-born neurons can contribute to hippocampal plasticity not only when they are young, but also as mature granule cells (Lemaire et al., 2012). We hypothesize that neurons produced during an animal's lifetime may build a neurogenic reserve that could be utilized in old age to partially compensate for reduced hippocampal neurogenesis. To test this hypothesis, we used immunohistochemistry to investigate neurogenesis in groups of rats ranging in age from 2.5 to 11 months that had run for a short period as juveniles. In this way, we can look at the long term effects of early exercise on neurogenesis, and how the different aspects of neurogenesis are regulated with aging.

## Methods

### Animals

**Figure 1:**
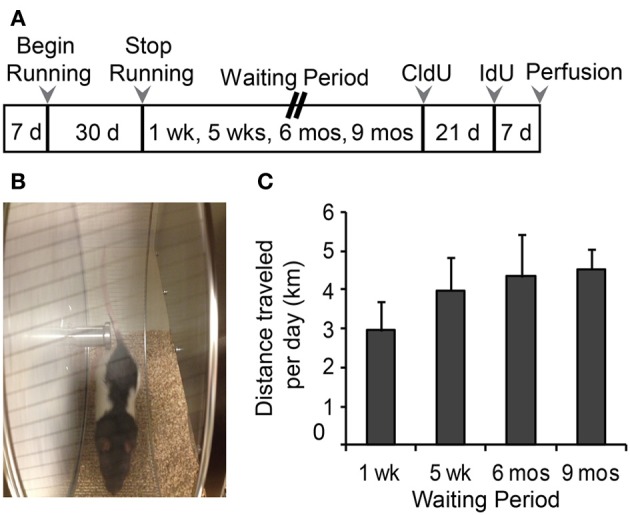
**(A)** Experimental time line. Male, Long Evans rats were 28 days old at the beginning of the study. Animals were divided into Runners (unlimited wheel access for 30 days) and Controls (non-runners). Four separate batches of Runners and Controls were perfused 1 week, 5 weeks, 6 months, and 9 months following the running period. Animals in the 1 week batch were injected with CldU only, at 7 days prior to perfusion. All remaining batches were injected with CldU at 4 weeks prior to perfusion, and with IdU at 7 days prior to perfusion. **(B)** Photograph of a 4 week old Male Long Evans rat on the running wheel apparatus. **(C)** Summary of running behavior (average daily running distance in km; mean ± s.e.m.) for all four batches. ANOVA showed no significant differences in the distance per day between Running cohorts (ANOVA, *P* = 0.519).

Four-week-old juvenile male Long Evans rats (Charles River, St Constant, Quebec, Canada), were maintained on a 12 h light/dark cycle with lights on between 0700 and 1900 h and remained individually housed for the duration of the experiment (Figure 1A). After a 1-week acclimatization period, rats were divided into two groups: Runners (*N* = 28 and Controls (sedentary non-runners; *N* = 24). Runners and Controls were maintained under the same housing conditions, including *ad libitum* access to food and water and basic standard cage toys, except that cages of Runners were fitted with a running wheel (circumference: 1.0706 m) (Figure 1B). Running wheels were equipped with a meter that attached to a computer, which monitored the speed, duration and daily running distance (VitalView Data Acquisition System, Mini Mitter a Respironics Company, Inc., Bend, OR, USA). As expected, rats predominantly ran during the dark phase of the cycle. After 30 days of unlimited wheel access, running wheels were removed from the cages of Runners. Runners and Controls were then subdivided into four groups, based on the time between cessation of running and perfusion. Cohorts of rats were perfused 1 week (*N* = 13), 5 weeks (*N* = 13), 6 months (*N* = 13), and 9 months (*N* = 13) after cessation of running. Therefore, in each cohort at the time of perfusion, rats were 10 weeks, 14 weeks, 8 months, and 11 months of age, respectively.

All animal procedures were in accordance with the National Institute of Health Guide for the care and use of laboratory animals, and the experimental protocol was approved by the Animal Care Committee at the University of Toronto.

### CldU and IdU administration

Thymidine analogs, 5-iodo-2′-deoxyuridine (IdU; 100357, MP Biomedicals, Solon, OH, USA) and 5-chloro-2′-deoxyuridine (CldU; 105478, MP Biomedicals) were administered intraperitoneally (ip) to all animals. CldU (85 mg/Kg dose) was dissolved at 10 mg/mL in saline with 0.5 μL/mL 10 N NaOH. IdU (115.5 mg/Kg dose) was dissolved at 10 mg/mL in saline with 4 μL/mL 10 N NaOH.

Four weeks prior to perfusion, all rats (except 10 week old group) received CldU injection. Rats that were 14 weeks and 8 months old rats received a single ip injection of CldU, while 11 month old rats received two ip injections of CldU 8 h apart to maximize uptake by dividing cells. In addition to CldU, animals received a single ip injection of IdU 1 week prior to perfusion (3 weeks post-CldU). Rats in the 10 week old group received CldU 1 week prior to perfusion instead of IdU. The experimental timeline is shown in Figure 1A.

### Tissue collection and processing

Animals were deeply anesthetized with isofluorane inhalation followed by transcardial perfusion with 300 mL ice-cold phosphate-buffered saline (PBS) followed by 300 mL ice-cold 4% paraformaldehyde (PFA). Following decapitation, brains were removed and placed in PFA for 24 h at 4°C. After post-fix, brains were placed in PBS containing 0.1% sodium azide, and stored until further processing.

### Blood sampling

Atrial blood was collected prior to perfusion in all animals. Blood was centrifuged, and stored at −20°C.

### Immunohistochemistry

Brains were cut in half, and the hippocampus was dissected from the right hemisphere in each animal. Dissociated hippocampi were sectioned along the dorso-ventral axis using a vibratome (VT1000S, Leica Microsystems, Heidelberg, Germany) into sections 30 μm thick. Sections were stored in PBS containing 0.1% sodium azide at 4°C. Twelve sections were selected from each animal using a systematic random sampling technique previously described (Wojtowicz and Kee, 2006).

All immunohistochemistry was conducted on free floating sections. Importantly, sections were rinsed extensively in PBS before processing and between each incubation. All primary and secondary antibody incubations were conducted in a PBS solution containing 0.3% Triton X-100. In experiments involving labeling of IdU and CldU sections were incubated at 45°C for 30 min in HCL (1.0 N) to denature DNA and unmask the antigen prior to incubation in primary antibody, preceded and followed by extensive rinsing.

#### Ki67

Single-label immunohistochemistry was used to identify Ki67, an endogenous marker of cellular proliferation (Scholzen and Gerdes, 2000; Kee et al., 2002). Briefly, sections were rinsed in PBS and incubated in rabbit polyclonal anti-Ki67 (1:200; VP-K451, Vector Labs, Burlingame, CA, USA) for 24 h at 4°C, followed by incubation in donkey anti-rabbit 568 (1:200, A11042, Life Technologies Inc., Grand Island, NY, USA) for 2 h at RT.

#### NeuN

All measurements (see analysis section Area and Length Measurements) were conducted on tissue that had been stained for NeuN, to label all neurons and provide clear anatomical boundaries within the hippocampus. NeuN was labeled using mouse monoclonal anti-NeuN (1:200; 24 h at 4°C; MAB377, Millipore, Billerica, MA, USA), followed by secondary antibody goat anti-mouse 488 (1:200; A11001, Life Technologies Inc.) for 2 h at RT.

#### Doublecortin (DCX) and IdU

Sections were double-labeled for IdU and doublecortin (DCX), a marker of immature neurons (Brown et al., 2003) in tissue from 14 week, 8 month, or 11 month old rats. In 10 week old rats (perfused 1 week after end of running period), sections were labeled for DCX and CldU. Sections were co-incubated in mouse anti-BrdU (1:700; 347580, BD Biosciences, Mississauga, ON, CA) or rat monoclonal anti-BrdU (1:1500; OBT0030, AbD Serotec, Biorad Laboratories, Inc., Raleigh, NC, USA) along with goat polyclonal anti-doublecortin (1:200; sc-8066, Santa Cruz Biotechnology, Dallas, TX, USA) for 24 h at 4°C. Sections were then co-incubated in donkey anti-mouse 594 (1:500; A21203, Life Technologies, Inc.) or donkey anti-rat 594 (1:200, A21209, Life Technologies, Inc.), along with donkey anti-goat 488 (1:200; A11055, Life Technologies, Inc.) for 2 h at RT.

#### Calbindin (CaBP) and CIdU

To identify cell survival and neuronal maturation of newly formed cells, double-label immunohistochemistry was conducted for CldU and Calbindin D-28 (CaBP). Sections from rats 14 weeks, 8 months, and 11 months of age were coincubated in primary antibodies rat monoclonal anti-BrdU (1:1500; Life Technologies, Inc.) and rabbit polyclonal anti-Calbindin D-28K (1:200; AB1778, Millipore) for 72 h at 4°C. Subsequently, sections were co-incubated in goat anti-rat 488 (1:1500, A11006, Life Technologies Inc.) and donkey anti-rabbit 568 (1:200; A11042, Life Technologies, Inc.) for 2 h at RT.

In all experiments, sections were mounted onto glass slides using double-distilled H_2_O (ddH_2_O), and coverslipped using PermaFluor (Thermo Scientific, Freemont, CA, USA). Immunohistochemical controls included the omission of primary antibodies, which resulted in lack of staining at the corresponding wavelength in each instance.

### Quantification and cell counts

Stereological assessment was used to count all single-labeled Ki67 and DCX-immunoreactive (ir) cells, using a fluorescent microscope (Nikon Optiphot-2; 20× dry lens). The counting region was defined as a 20 μm-wide region centered at the SGZ, and bordering the GCL and hilus of the DG (Wojtowicz and Kee, 2006). Absolute numbers were counted in both the supra and infrapyramidal blades, with consistent parameters. The average number of cells per section was multiplied by the number of hippocampal sections to yield the estimated total number of cells per DG in each animal. Regional analyses were also conducted, with the number of cells per section in dorsal, medial, and ventral hippocampus calculated, as previously described (McDonald and Wojtowicz, 2005).

All dual-labeled sections were counted stereologically using a Leica TCS-SL laser-scanning confocal microscope (Leica Microsystems) using a 40× oil immersion objective lens. In sections dual-labeled for DCX/IdU, numbers of single IdU and dual DCX/IdU cells were counted in the SGZ and granule cell layer (GCL) of the DG, along the dorsal-ventral axis using the same stereological parameters as described above. In tissue dual-labeled for CldU and CaBP, numbers of single CldU, and dual-labeled CaBP/CldU cells were counted within the GCL (both supra and infrapyramidal blades) of each section. The average numbers of single and dual-labeled cells per section was calculated, and then multiplied by the total number of DG sections in each animal to obtain the total number per DG. The percentage of IdU and CldU cells expressing DCX or CaBP was calculated for each animal. Cells were considered dual-labeled if a BrdU-positive nucleus (identifying presence of IdU or CldU) was surrounded by a CaBP-ir or DCX-ir cytoplasm. Orthogonal views confirmed colocalization in the same neurons in 1 μm confocal sections.

### Area and length measurements

Area and length measurements were taken from a subset of animals in each group (*N* = 4 Runners, *N* = 4 controls in each cohort), using single-labeled NeuN staining to delineate hippocampal sub-regions. Images of NeuN-labeled hippocampal sections were taken using a Nikon Optiphot-2 fluorescent microscope equipped with a Sensicam CCD camera. All images were imported into Image J software where measurements were conducted. Surface area measurements of CA1, DG (including the hilus, GCL and molecular layer), and SGZ length was also measured in the same sections. To calculate volume for the DG, GCL and CA1, average surface area for each region within each animal was multiplied by the number of DG sections per animal and the thickness of each section (30 μm).

### Statistical analyses

Differences between age groups, as well as between Runners and Controls within each age group for immunohistochemistry were analyzed using Two-Way ANOVAs (Age × Group), to investigate main effects of age, group (Runners vs. Controls), and Age × Group interactions. All pairwise multiple comparisons for the Two-Way ANOVA analyses were conducted using the Holm–Sidak method. One-Way ANOVAs were conducted for running data analyses (cumulative distance and average daily running distance) within cohorts, and pairwise comparisons were conducted using Tukey's test. *T*-tests were used to compare running data from the first and last 15 days of the running period. Statistical significance was considered as *P* < 0.05.

#### Exponential regression analysis

The decay of neurogenesis with age is not a linear process. Instead, it can be adequately fitted with a single exponential curve (Wojtowicz, 2011). The numbers of Ki67, DCX, CaBP/CldU, and DCX/IdU cells were plotted as a function of age in Runners and non-running Controls. Analysis was performed using SigmaPlot 12.5 software. The exponential parameters a (the initial value) and b (rate constant of decay) are informative in the analysis of the decay process.

## Results

### Running data

Running distances were monitored daily as well as the time of day that each animal spent running. The data shows that the rats predominantly ran during the dark phase of their cycle. Cumulative running distance or average distance traveled per day did not differ between cohorts (Figure 1C), and across the entire running period of 30 days, rats ran an average of 3.96 ± 0.35 km/day and 118.79 ± 10.59 km in total.

To further investigate the running trajectories in each of the cohorts across the 30 day running period, running data was divided into 4 weekly segments (days 1–7, 8–14, 15–21, 22–30). One-Way ANOVAs revealed between groups effects for each of the cohorts [1 week: *F*_(2, 26)_ = 21.393, *P* < 0.001; 5 week: *F*_(3, 26)_ = 4.961, *P* = 0.007; 6 months: *F*_(3, 26)_ = 23.651, *P* < 0.001; 9 months: *F*_(3, 26)_ = 3.212, *P* = 0.039]. Pairwise comparisons indicate that rats in the 1 week and 6 month cohorts ran significantly less in their first 7 days of running wheel access compared to days 15–21 (1 week, *P* = 0.016; 6 month, *P* < 0.001) and 22–30 (1 week and 6 months, *P* < 0.001). To a similar extent, rats in the 5 week (14 weeks old) and 9 month (11 months old) cohorts ran significantly less in their first week of wheel access compared to 15–21 of wheel access (5 week, *P* = 0.004; 9 month, *P* = 0.025).

Corticosterone levels were measured in atrial blood taken just prior to perfusion, and did not differ between groups at any time point.

### Area and length measurements using NeuN staining

Single-labeled NeuN was used to label neurons in hippocampal sections. Area and volume measurements are shown in Table 1. Comparisons between runners and controls did not differ in any area or volume measurements for the DG, GCL, and CA1. Two-Way ANOVAs revealed age-related changes in area measurements for all regions examined (main effect of age, *P* < 0.001). Pairwise comparisons showed that 11 month old rats had significantly greater DG, GCL, and CA1 area than rats 14 weeks of age, and greater CA1 area than both 14 week and 8 month old rats, regardless of whether the animals were runners or controls.

**Table 1:** **Area (mm^2^) and volume (mm^3^) measurements**.

**Cohort**	**Area (mm^**2**^)**			**Volume (mm^**3**^)**		
	**DG**	**GCL**	**CA1**	**DG**	**GCL**	**CA1**
**5 WEEK**						
Control	2.18 ± 0.10	0.35 ± 0.02	1.03 ± 0.07	14.85 ± 0.43	2.39 ± 0.10	7.05 ± 0.44
Runner	2.32 ± 0.09	0.38 ± 0.02	1.04 ± 0.03	16.32 ± 0.63	2.64 ± 0.12	7.32 ± 0.21
**6 MONTH**						
Control	1.96 ± 0.10^#^	0.34 ± 0.01	1.10 ± 0.09	13.43 ± 0.63^#^	2.32 ± 0.08^#^	7.54 ± 0.57
Runner	1.84 ± 0.05^#^	0.31 ± 0.01	1.02 ± 0.06	12.09 ± 0.31^#^	2.06 ± 0.09^#^	6.70 ± 0.31
**9 MONTH**						
Control	2.03 ± 0.04^#^	0.31 ± 0.01^#^	1.35 ± 0.02^#^^,^^§^	13.00 ± 0.48^#^	2.00 ± 0.67^#^	8.63 ± 0.26^#^^,^^§^
Runner	2.03 ± 0.05^#^	0.31 ± 0.01^#^	1.44 ± 0.05^#^^,^^§^	12.22 ± 0.78^#^	1.89 ± 0.11^#^	8.63 ± 0.53^#^^,^^§^

# Age effect, significantly different from 5 week cohort;

§ *Age effect, significantly different from 6 month cohort*.

The calculated volumes were compared between Runners and Controls in each age group, to assess the possibility that difference in cell counts could be attributed to differences in the size of the GCL, DG or CA1. Our results showed no significant effect of group in any cohorts; DG, GCL and CA1 volumes did not differ between Runners and Controls in any age group.

### Cellular proliferation: Ki67

**Figure 2:**
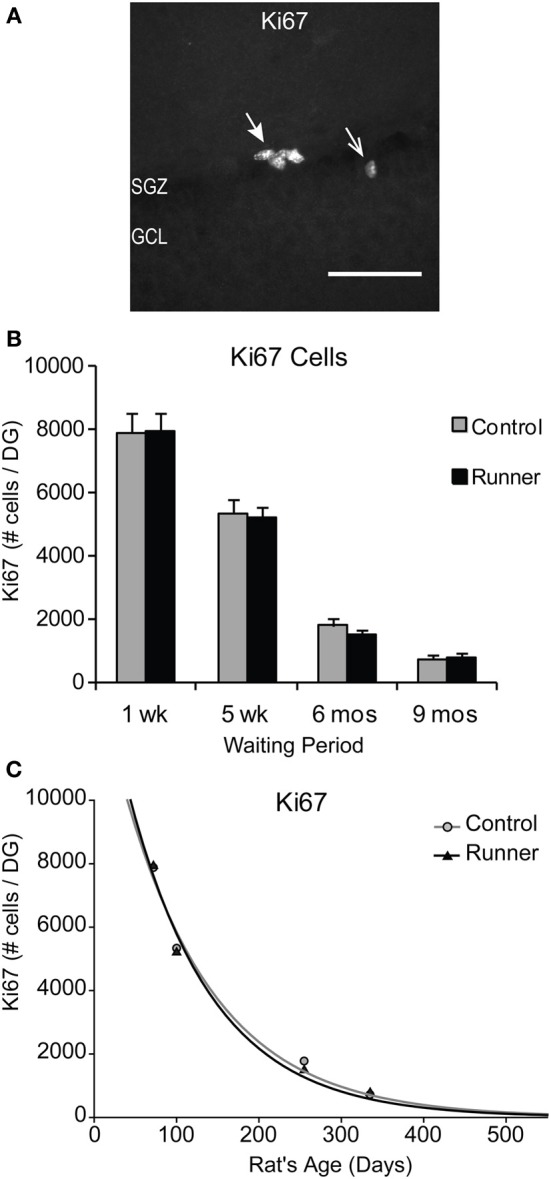
**Ki67 cells in the rat DG. (A)** Image shows typical Ki67 labeling of proliferating precursors in the subgranular zone (SGZ) bordering between Hilus and granule cell layer (GCL). Full arrowhead indicates one cluster of several nuclei and open arrowhead indicates one single nucleus. Scale bar, 50 μm. **(B)** Numbers (mean ± s.e.m.) of Ki67 cells per DG. No significant differences were found between Runners and Controls in any cohort (Two-Way ANOVA, *P* = 0.969). **(C)** Exponential decay curve fitted to the data for controls (circles) and runners (triangles) show the decay of proliferation with age. In this and subsequent figures the curves were fitted using regression procedure and were significant at *P* < 0.05. The two parameters were a = 14.376 and b = 0.0090 in Controls and a = 15.418 and b = 0.0098 in Runners. Thus, there were no significant differences between the initial cell numbers (a) or rate constants (b).

Immunohistochemical labeling for the endogenous marker Ki67, was used to identify proliferating cells at the time of perfusion (Kee et al., 2002). Examples of Ki67-ir cells are shown in Figure 2A. There were no differences in the number of Ki67-labeled cells per section or per DG between Runners and Controls in any of the age groups (Figure 2B). Not surprisingly though, there were significant age-related declines in cellular proliferation; absolute numbers of Ki67 cells per section [*F*_(3, 42)_ = 118.212, *P* < 0.001] and DG [*F*_(3, 42)_ = 134.66, *P* < 0.001] were significantly decreased as a function of age. These data were also fitted with exponential curves using a regression procedure (Wojtowicz, 2011). There were no significant differences between the initial cell numbers (*a*) or rate constants (*b*). The two parameters were *a* = 14.376 ± 2041 (SE) and *b* = 0.0090 ± 0.0015 (SE) in Controls and *a* = 15.418 ± 2528 (SE) and *b* = 0.0098 ± 0.0018 (SE) in Runners, and show that the rates of decay were not different between Runners and Controls (Figure 2C).

In addition, there were no regional differences in the number of Ki67-labeled cells in dorsal, medial, or ventral sections. To determine whether differences were present in numbers of cells between the supra and infrapyramidal blades, and to control for differences in the size of each blade, numbers of Ki67 cells were standardized per mm SGZ length. After standardization for length of each blade, Runners in the 5 week group (14 weeks of age) showed significantly more Ki67- ir cells per mm lower blade compared to the upper blade (*P* = 0.015).

Next, to determine whether in these rats there is an association between the amount of running and number of new cells produced, we used cumulative distance ran (km) over the 30 day period and absolute numbers of Ki67 cells per DG. Results showed no significant correlation between distance ran and amount of cellular proliferation.

### Neuronal differentiation: IdU and DCX

**Figure 3:**
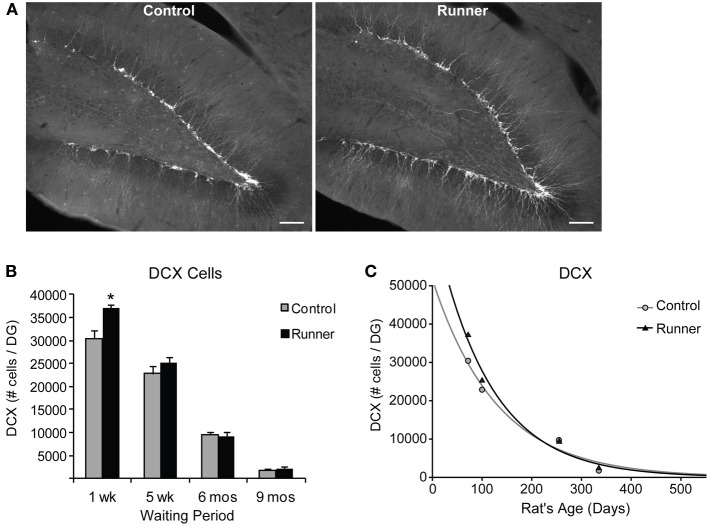
**Neuronal differentiation in the rat DG. (A)** Fluorescent microscopic images (10×) showing doublecortin (DCX) cells in Runners and Controls at 1 week post-running (10 weeks of age). Scale bar, 100 μm. **(B)** Number (mean ± s.e.m.) of DCX cells per DG. At 1 week post-running (10 weeks old), Runners had significantly more DCX cells per DG than Controls (Two-Way ANOVA, ^*^P < 0.001). No significant differences were detected between Runners and Controls at any other time point. **(C)** Exponential decay curve for DCX shows parameters a = 51.650 and b = 0.0077 in Controls and a = 67.680 and b = 0.0089 in Runners. This is consistent with the higher initial cell number at younger ages and slightly faster decay in runners compared with controls.

To identify newly generated cells (≤1 week old) that had progressed to an immature neuronal phenotype, immunohistochemical labeling for IdU and DCX was conducted. Animals in all age groups received IdU 1 week prior to perfusion, so that this aspect of neurogenesis could be analyzed within Runners and Controls of different age groups in parallel. This 1 week time point corresponds to optimal visualization of DCX protein, which shows peak expression in newly differentiated neuronal cells ~7 days of age (McDonald and Wojtowicz, 2005). First, to investigate differences in overall differentiation of adult-born cells to a neuronal phenotype, single-labeled DCX was counted in all animals (Figure 3A). Two-Way ANOVA revealed a significant Age × Group interaction [*F*_(3, 42)_ = 4.720, *P* = 0.006], and pairwise comparisons demonstrated that Runners showed significantly more DCX cells per section and cells per DG compared to non-running Controls (*P* < 0.001) in 10 week old rats, but not in any other age group (Figure 3B). There was also a significant main effect of age [*F*_(3, 42)_ = 381.502, *P* < 0.001], and pairwise comparisons showed that all groups were significantly different from one another (*P* < 0.001 for all comparisons). The number of DCX-expressing cells in rats 6 months (8 months old) and 9 months (11 months old) post-running rats was only 6% and 6% of the number in 10 week old rats (perfused 1 week after running), indicating a 72% and 94% decrease, respectively, (*P* < 0.001). The exponential decay curve shows parameters *a* = 51.650 ± 7414 (SE) and *b* = 0.0077 ± 0.0014 (SE) in Controls and *a* = 67.680 ± 11,241 (SE) and *b* = 0.0089 ± 0.0018 (SE) in Runners. The exponential fit data suggests that the initial increase seen in runners at 1 week post-running (10 weeks old) recovers with a faster decay rate in the running cohort (Figure 3C).

In these experiments, the total number of IdU-labeled cells reflects cells that have survived 1 week since injection, and includes both cells that have differentiated to neuronal and non-neuronal phenotypes. Results indicated a significant effect of Group for IdU-labeled cells [*F*_(1, 37)_ = 4.295, *P* = 0.045], and pairwise comparisons revealed that 10 week old Runners (perfused 1 week after running) had significantly more IdU-labeled cells than Controls at that age (*P* = 0.007). There were no significant differences between Runners and Controls in any other age group (data not shown).

**Figure 4:**
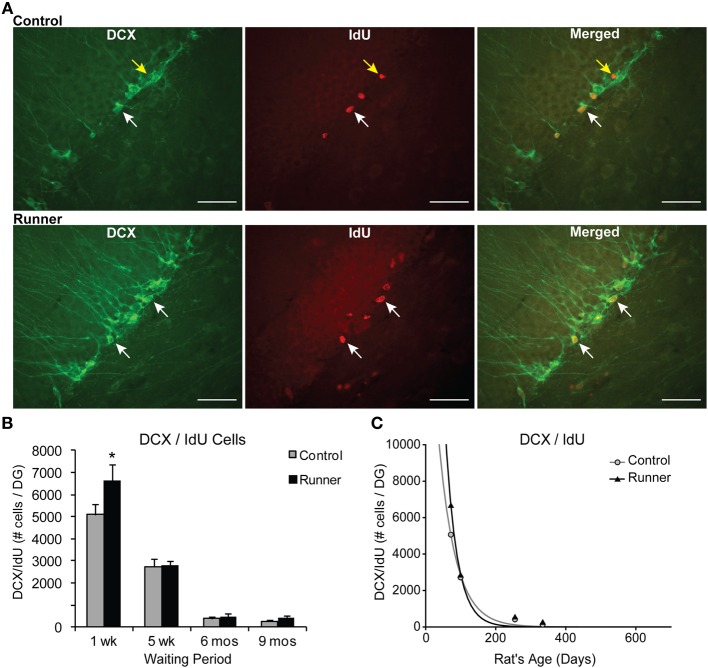
**Neuronal differentiation and survival in 1 week old neurons. (A)** Confocal microscopic images (40×) showing DCX and CldU labeled cells in the DG of 10 week old Control (*top panels*) and Runner (*bottom panels*). White arrows indicate double-labeled cells, and yellow arrow indicates single-labeled CldU cell. Scale bar, 50 um. **(B)** Numbers (mean ± s.e.m.) of dual-labeled DCX/IdU cells. Runners in the 1 week cohort (10 weeks of age) showed significantly more dual-labeled cells than Controls (Two-Way ANOVA, ^*^P = 0.007). There were no differences between Runners and Controls in any other group. **(C)** Exponential decay curve for DCX/IdU shows parameters a = 22.120 and b = 0.0206 in Controls and a = 58.070 and b = 0.0301 in Runners. The data from the regression analysis is consistent with higher initial cell numbers in Runners early on, but faster decay with age.

The percentage of IdU cells expressing DCX showed that a large majority ~85% of IdU cells express DCX across all age groups in both Runners and Controls (Range: 75–92%), and no significant differences between Runners and Controls were detected. Examples of dual-labeled cells are shown in Figure 4A. Absolute numbers of dual-labeled DCX/IdU cells were significantly different between 10 week old Runners and Controls (*P* = 0.003). This effect was not present in any other age group (Figure 4B). An exponential decay curve fitted to the DCX/IdU data shows parameters *a* = 22.120 ± 5255 (SE) and *b* = 0.0206 ± 0.003 (SE) in Controls and *a* = 58.070 ± 21,676 (SE) and *b* = 0.0301 ± 0.0048 (SE) in Runners. This is consistent with the greater numbers of cells at 1 week post-running (10 weeks of age) in Runners, but more rapid decay with age (Figure 4C).

The data showed significant main effects of age for total IdU [*F*_(3, 37)_ = 129.368, *P* < 0.001] and dual DCX/IdU [*F*_(3, 37)_ = 124.04, *P* < 0.001]. Pairwise comparisons showed the 10 and 14 week old animals had significantly greater numbers of cells compared with all other age groups (*P* < 0.001 for all comparisons), regardless of whether they were Runners or sedentary Controls. Animals 8 and 11 months of age did not differ significantly from one another.

Regional distribution was also examined, with no significant differences between Runners and Controls at any age group in dorsal, medial, or ventral sections.

Finally, to determine if early running increased dendritic growth and morphological integration of adult born neurons, DCX staining was used to analyze dendritic branching as described previously (Rosenzweig and Wojtowicz, 2011; Whissell et al., 2013). Tissue from Runners and Controls in each age group was used for these analyses, and absolute numbers of DCX-labeled cells were counted, as well as the numbers of primary and secondary dendrites in the middle GCL and outer edge of the GCL, respectively, Rosenzweig and Wojtowicz (2011); Whissell et al. (2013). The level of primary dendrite branching was determined by calculating the ratio of primary dendrite crossings to numbers of DCX-labeled cells, and the level of secondary branching was calculated as a ratio of secondary dendrites to primary dendrites (Rosenzweig and Wojtowicz, 2011; Whissell et al., 2013). Our analyses did not reveal any significant differences in absolute numbers of dendrites, or dendritic branching in the GCL between Runners and Controls in any age group. There were however, significant age-related declines in the number of primary and secondary dendrites and in the level of primary branching (Two-Way ANOVA, *P* < 0.001). Pairwise comparisons revealed that significant age-related declines in numbers of primary and secondary dendrites were present between all age groups except 8 month and 11 month rats (*P* < 0.001). Finally, the level of primary branching was significantly greater in 10 week old rats than all other age groups (*P* < 0.01).

### Cell survival and maturation: CldU and CaBP

To investigate cell survival, CldU was injected 4 weeks before perfusion in 14 week, 8 month, and 11 month old rats, which were sacrificed 5 weeks, 6 months, and 9 months after cessation of running, respectively. Therefore, within each age group, all CldU cells examined were the same age (4 weeks old) and enabled us to look at this maturational stage correspondingly. CaBP was immunostained alongside CldU, to allow visualization of mature neurons (McDonald and Wojtowicz, 2005; Wojtowicz and Kee, 2006).

The total numbers of CldU cells were counted in sampled sections and represented cells (neurons and non-neurons) that had survived 4 weeks. Two-Way ANOVA showed a significant Age × Group interactions for both numbers of total CldU per DG [*F*_(2, 29)_ = 6.872, *P* = 0.004] and number of total CldU cells per section [*F*_(2, 29)_ = 4.208, *P* = 0.025]. Pairwise comparisons revealed that only the 14 week old Runners (5 week waiting period) had significantly greater numbers of cells than non-running Controls (*P* < 0.001). However, there were significant age-related declines in the number of surviving cells across the age groups. The number of surviving cells in 8 month old rats showed an 89% decline in the numbers of CldU cells (*P* < 0.001). Similarly, in rats 11 months of age, the number of CldU cells was only 8% of the number in 14 week old rats, indicating a 92% decline (*P* < 0.001). Although there was a 29% decrease in cell survival between 8 and 11 month old rats, it was not a statistically significant difference (*P* = 0.70).

**Figure 5:**
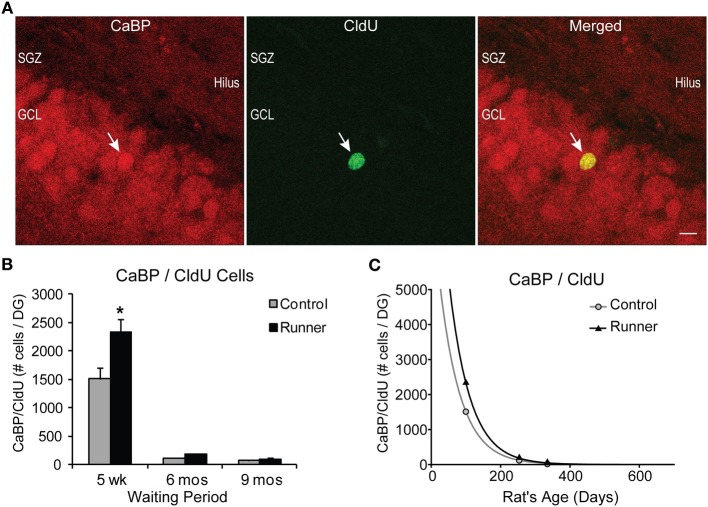
**Survival and neuronal maturation. (A)** Confocal microscopic images (40×, 1 μm thickness) showing CaBP and CldU-labeled cells in the GCL. White arrow indicates dual-labeled CaBP/CldU cell in the GCL. Scale bar, 10 um. **(B)** Number (mean ± s.e.m.) of dual-labeled cells per DG. Runners in the 5 week cohort (14 weeks of age) showed significantly more dual-labeled cells than Controls (Two-Way ANOVA, ^*^P < 0.001). **(C)** Exponential decay curve for CaBP/CldU shows parameters a = 7775 and b = 0.0164 in Controls and a = 12.233 and b = 0.0166 in Runners. Regression analysis suggests substantially higher initial cell number in runners but virtually identical decay rates in runners and controls. Hence, persistent change in cell maturation and survival with aging.

The percentage of CldU cells colocalizing CaBP did not differ between Runners and Controls for any age group, however there were significant effects of age. Examples of CldU cells coexpressing CaBP is shown in Figure 5A. At 5 weeks post-running (14 week old), a large majority (79%) of CldU cells coexpressed CaBP in Runners and Controls, which was significantly different from the percent colocalization at 6 months (54%; 8 months old) and 9 months (43%; 11 months old) post-running (*P* < 0.001).

Absolute numbers of dual-labeled CaBP/CldU cells showed significant Age × Group interactions for both cells per DG [*F*_(2, 29)_ = 6.418, *P* > 0.001] and cells per section [*F*_(2, 29)_ = 5.435, *P* = 0.010] (Figure 5B). Pairwise comparisons revealed that 5 weeks post-running (14 weeks old), Runners had significantly more dual-labeled cells than non-running Controls (*P* < 0.001), and there were no differences between Runners and Controls in the other age groups (Figure 5B).

Results also revealed a significant age-related decline in the numbers of dual-labeled CaBP/CldU cells, with 14 week old rats showing significantly more dual-labeled cells than rats at both 8 and 11 months of age (*P* < 0.001 for both comparisons), which represented a decrease of 92% (*P* < 0.001) and 95% (*P* < 0.001), respectively (Figure 5B).

Exponential decay curves fitted to the data with parameters *a* = 7775 ± 175 (SE) and *b* = 0.0164 ± 0.0086 (SE) in Controls and *a* = 12.233 ± 130 (SE) and *b* = 0.0166 ± 0.0001 (SE) in Runners, determine that the rates of decline were almost identical between Runners and Controls (Figure 5C), despite the initial increase in numbers of cells in Runners at 14 weeks. These regression analyses demonstrate that the processes of cell survival and maturation are qualitatively different from all other parameters measured thus far. With nearly identical rates of decline, the differences between Runners and Controls early on are preserved across the life span.

Finally, we saw instances of CldU cells in the hilus region of the DG in some animals, and we did not observe any CaBP expression within those cells.

## Discussion

In the present study, the effects of running on various facets of neurogenesis were investigated over both short and long timescales in cohorts of rats that were sacrificed 1 week, 5 weeks, 6 months, and 9 months following a running period of 30 days as juveniles. Results from this study demonstrate the remarkable capacity for homeostatic regulation of neurogenesis. After short term enhancement induced by running, the total numbers of differentiated young neurons, and a subpopulation of 1 week old differentiated neurons (IdU and DCX dual labeling) were restored to Control (non-Runner) levels by the time rats reached 3 months of age. However, one aspect that was increased in Runners and did show long term alterations was the process of neuronal survival/maturation.

Due to the long duration of our study we were able to evaluate not only different aspects of neurogenesis such as proliferation, differentiation and survival/maturation, but also how these processes change with aging. Remarkably, our findings show the rate of decay of the CaBP/CldU double-labeled neurons with age was almost identical in Controls and in Runners even though the initial values shortly after running and were 50% greater in the latter (Figure 5B). The consequence is the preservation of the running-induced changes into maturity and old age. An important feature of the design of this study is that the age of the adult-born neurons used to assess differentiation, survival and maturation, correspond across all groups. For example, regardless of the age of the animal, all cells used to assess maturation and survival were 4 weeks old.

To our knowledge, this is the first study to examine the effects of exercise over an extended time course, up to 9 months after animals stopped running. Other studies have looked at variable running times (long term or short term running in both young, middle age and old age rodents) (Naylor et al., 2005; Van Praag et al., 2005; Wojtowicz et al., 2008; Gebara et al., 2013), but the interval between cessation of running and BrdU injection or neurogenesis investigation have been relatively short. The design of our study allowed us the ability to look at long term effects of running from a transient period in early life that extend into late middle age.

Adult neurogenesis is functionally regulated in two stages: “GABA phase,” in which GABA acts to depolarize immature neurons, and “Glutamate phase,” in which neurons are depolarized through the actions of glutamate. In between these stages, there is a period of combined effects, when GABA provides depolarization and activates the initially silent NMDA-only synapses (Chancey et al., 2013). After 3 weeks, GABA depolarization is phased out, and AMPA glutamatergic synapses are phased in (Chancey et al., 2013). This transition from GABA only signaling to glutamatergic synapse formation is not all-or-none, and is presumably activity-dependent (Kee et al., 2007; Tashiro et al., 2007; Kitamura et al., 2010). It has been previously speculated that GABAergic depolarization plays a key role in AMPA insertion (Ben-Ari et al., 1997), and has been recently shown that the depolarizing action of GABA unsilences NMDA-containing synapses and promote AMPA insertion on developing neurons (Chancey et al., 2013). If this process is activity-dependent, then there may be many NMDA-only synapses on relatively mature neurons that have not undergone this transition, essentially “in waiting.”

Recent evidence in rats has shown that even several months after birth, mature adult-born neurons can be selectively recruited and shaped by learning tasks (Lemaire et al., 2012). This activation is associated with the formation of dendritic spines and extension of dendrites (Lemaire et al., 2012), and suggests that even after adult formed neurons have undergone critical periods of survival and plasticity, and have existed as mature granule cells for several months, they are still responsive to learning tasks and remain highly plastic. This is strong support for the idea that experience-dependent plasticity is not transient for adult-born dentate granule cells, but extends into maturity. This study is distinguished from the present study in that Lemaire et al. (2012) examined adult-born cells that were several (2–4) months of age, while in our study using aging rats, age of the neurons used to assess maturation and survival were the same despite the animal's age.

The present study has addressed the state of maturation of newborn granule cells at 4 weeks after birth in different aging cohorts. This 4 week time point should correspond to neurons that have transitioned to predominantly glutamatergic excitatory control (Ambrogini et al., 2004), and exhibit enhanced synaptic plasticity (Ge et al., 2007). The availability of neurons at this time point is relevant for both neurons that are ready to be recruited immediately and neurons that may be kept in reserve, mature and recruitable when behavioral need arises. This stage of development cannot be detected by counting neurons at the proliferative stage (i.e., Ki67) or immature stage when DCX is expressed. Yet, the increased number of these neurons “in waiting,” provides a valuable functional reserve. The presence of such a reserve would be consistent with the notion that a relatively small number of new neurons in aging animals may be functionally significant since learning behaviors recruit relatively small numbers of dentate granule cells (Chawla et al., 2005). Therefore, we propose that despite age-related decline in neurogenesis, there are still a relatively large number of newly formed and highly plastic mature neurons that survive in the GCL and are available to be recruited and structurally modified by learning. This may constitute a functional neurogenic reserve, and the concept is supported by data showing generally sparse activation of dentate granule neurons observed *in vivo* during learning tasks (Chawla et al., 2005; Piatti et al., 2013). Future studies investigating the effects of early running on selective recruitment of these adult-born, mature neurons to learning tasks (using immediate early gene markers) will be useful for our understanding of their functional significance for incorporation into memory circuits.

Previous studies using rodents have shown that the effects of running to increase cellular proliferation are evident after only a few days of running (Naylor et al., 2005; Kronenberg et al., 2006; Van Praag, 2008), but that after longer periods of running, this pro-proliferative effect is not evident (Naylor et al., 2005; Kronenberg et al., 2006; Wojtowicz et al., 2008). This may explain why we have not observed any effects of running on proliferation even at short survival times, e.g., 1 week cohort. These results also suggest that there are no long term changes in the proliferating pool of stem cells giving rise to new neurons in the old age. Other changes in life style such as enriched environment could be more effective in this regard.

In the present study, a running period of 30 days in juvenile (1 month old) rats is arbitrary and it is possible that a longer period of exercise (i.e., 2 months or more), or intermittent exercise for a longer period is sufficient to induce even more pronounced long term changes. However, it is possible that early in life there is a critical period where the potential to build the neurogenic reserve and have it last a lifetime (persist with aging) is greatest.

An interesting question relating to this work that may be addressed in future studies is whether animals who experienced a period of exercise would show a greater response to another period of exercise later in life than sedentary animals. There is data showing that aged mice can respond to exercise with increased neurogenesis (Van Praag et al., 2005), but the outstanding question is whether a history of exercise would result in even greater increases in neurogenesis, and longer lasting persistent effect (Kempermann et al., 2010).

Age-related changes are an important theme in our study, and one consistent finding was that considerable age-related declines in neurogenesis (proliferation, differentiation, survival/maturation) were present regardless of whether animals had been exercised or not. These included rats ranging in age from 2.5 months (10 weeks) to 11 months of age, and our findings are in line with previous work showing age-related declines in neurogenesis in rats (Seki and Arai, 1995; Kuhn et al., 1996; McDonald and Wojtowicz, 2005; Lee et al., 2012). Age-related declines may be due to a variety of factors that can influence neurogenesis, including elevated circulating cytokines (Monje et al., 2003; Keohane et al., 2010; Villeda et al., 2011), and increased numbers of microglia in the hippocampus (Kohman et al., 2012; Gebara et al., 2013). Aging is associated with increased circulating glucocorticoids, which are also implicated in suppression of neurogenesis (Cameron and McKay, 1999; Montaron et al., 1999), vascular changes (Black et al., 1989; Sonntag et al., 1997) and release of different growth factors (Sonntag et al., 1997; Aberg et al., 2000; Lichtenwalner et al., 2001), which all likely play an important role in the regulation of neurogenesis. In a large number of studies addressing these various factors with aging, these effects have been improved with exercise (Van Praag et al., 2005; Wu et al., 2008; O'Callaghan et al., 2009; Creer et al., 2010; Kohman et al., 2012; Marlatt et al., 2012; Gebara et al., 2013; Speisman et al., 2013) (For review, see Voss et al., 2013). Thus, exercise can modulate a variety of factors that are important for learning and play important roles in regulating neurogenesis.

Increased BDNF has long been shown to be associated with exercise in the DG of rodents. Neeper et al. (1995) was the first to show that exercise increases BDNF mRNA in the hippocampus (Neeper et al., 1995). Since then, a large number of studies have demonstrated that BDNF is upregulated in the hippocampus with exercise, whether it is continual, intermittent, short, or long term (Neeper et al., 1995, 1996; Berchtold et al., 2005; Ding et al., 2011). More specifically, BDNF is increased within the DG and not in other hippocampal areas like CA1 (Farmer et al., 2004; Vaynman et al., 2004). The effects of exercise to increase BDNF are still present in aged animals, but to a lesser extent; exercise augments BDNF levels less effectively in older animals (Adlard et al., 2005).

A common theme in Alzheimer's disease (AD) and cognitive or neurogenic reserve literature is the idea that early intervention may be a key therapeutic strategy (Kempermann, 2008; Mu and Gage, 2011). Based on findings that alterations in neurogenesis take place before the onset of AD hallmarks (Demars et al., 2010; Krezymon et al., 2013), potentiating neurogenesis through exercise may be a useful strategy to combat the development and etiology of the disease (Um et al., 2008; Demars et al., 2010; Garcia-Mesa et al., 2011; Krezymon et al., 2013). Using a mutant mouse model, Demars et al. (2010), showed that proliferation and differentiation were decreased by as early as 2 months of age, months before neuroanatomical hallmarks of AD present themselves (Demars et al., 2010). Similarly, findings from a study using a different transgenic model (Tg2576) showed altered neurogenesis (cell survival, maturation, morphology) in young mice that preceded AD pathology (Krezymon et al., 2013). In a recent study using an AD mouse model, exercise was shown to restore hippocampal neurogenesis (Rodriguez et al., 2011). Furthermore, the benefits of exercise on transgenic AD mouse models extend to other pathways, which may converge with regulators of neurogenesis. These include effects of exercise to decrease AD pathology and inflammatory factors, and increase plasticity and BDNF (Adlard et al., 2005; Um et al., 2008; Nichol et al., 2009; Yuede et al., 2009; Garcia-Mesa et al., 2011; Liu et al., 2011). Based on our current findings, it is reasonable to hypothesize that early exercise may reduce some of the cognitive impairments associated with these AD mouse models by augmenting early neurogenic changes that take place in the development of AD.

In summary, this study demonstrates the remarkable homeostatic regulation of neuronal differentiation in response to exercise and with aging. In a classical argument where homeostasis contrasts with plasticity we can offer a compromise where the levels of neurogenesis are strictly controlled during aging, but the functional regulation of neuronal survival and differentiation of newly-born neurons can be enhanced. Early exposure to exercise may produce a functional reserve of adult-born neurons that may be available for recruitment to learning tasks. These findings are important for our understanding of the modulation of neurogenesis by exercise with age, and highlight the contention that early exercise may be a key component of healthy aging.

### Conflict of interest statement

The authors declare that the research was conducted in the absence of any commercial or financial relationships that could be construed as a potential conflict of interest.
